# Post-ERCP Emphysematous Cholecystitis in a Young Woman: A Rare and Potentially Fatal Complication

**DOI:** 10.1155/2017/1971457

**Published:** 2017-03-21

**Authors:** Roisin Stack, Joseph McLoughlin, Amy Gillis, Barbara M. Ryan

**Affiliations:** ^1^Department of Gastroenterology, Tallaght Hospital and Trinity College, Dublin, Ireland; ^2^Department of Surgery, Tallaght Hospital and Trinity College, Dublin, Ireland

## Abstract

A 45-year-old woman with suspected Functional Biliary Sphincter Disorder (FBSD) developed* Clostridium perfringens* related emphysematous cholecystitis after ERCP. A low index of suspicion for emphysematous cholecystitis in this young, otherwise healthy woman led to a significant delay in making the correct diagnosis, and air in the gallbladder was wrongly attributed to a possible gallbladder perforation. ERCP is associated with significant risks, particularly in patients with FBSD, where diagnostic uncertainty renders the balance of risk versus benefit even more critical. Post-ERCP emphysematous cholecystitis secondary to* Clostridium perfringens* is a rare but potentially fatal complication.

## 1. Introduction

It is well established that endoscopic retrograde cholangiopancreatography (ERCP) is associated with significant risks. However emphysematous cholecystitis secondary to* Clostridium perfringens* infection is exceedingly rare after ERCP and there are only four such reports in the literature. Emphysematous cholecystitis is generally thought to be an infection of older patients with significant comorbidities and not of young, otherwise healthy individuals.

## 2. Case Report

A 45-year-old, woman, with a three-year history of severe, intermittent upper abdominal pain, underwent an ERCP and sphincterotomy for suspected Functional Biliary Sphincter Disorder (FBSD) or what would previously have been classified as Sphincter of Oddi Dysfunction (SOD) type II. She was otherwise well with no significant comorbidities. She had previously been extensively investigated: ultrasound, cross sectional abdominal imaging, upper GI endoscopy, endoscopic ultrasound, HIDA scan, and extensive bloods all were normal, apart from intermittent, mildly elevated LFTs. Biliary manometry was not performed. A clinical diagnosis of suspected FBSD was made on the basis of the biliary character of the pain and mildly raised LFTs on a number of occasions. The patient had failed to respond to trials of a number of medications for almost two years. Following extensive discussion and consideration, a decision was made to proceed to elective ERCP.

The patient was given prophylactic 3rd generation cephalosporin preprocedure in line with current guidelines. The ERCP was an uneventful procedure and there was no instrumentation to the pancreatic duct. The CBD was of normal diameter and a sphincterotomy was performed. 100 mg of diclofenac was given PR immediately after procedure. The patient developed upper abdominal pain several hours after procedure and on examination she was mildly tender in the right upper quadrant and epigastrium. She was admitted overnight for observation, was given IV fluids, and was kept fasting. Bloods the following morning were normal apart from a mildly raised CRP of 12 mg/L. The pain had eased and the patient, who was keen to go home, was discharged.

**Figure 1:**
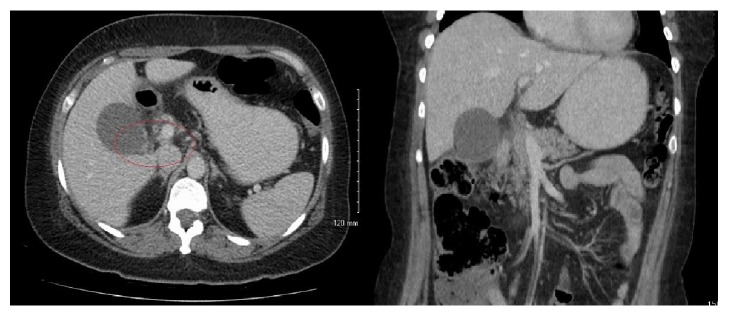
CT abdomen pelvis day 2: periportal and pericholecystic fat stranding with hyperaemia of the extrahepatic bile duct and adjacent stranding. Findings are suspicious for an ascending cholangitis and cholecystitis.

**Figure 2:**
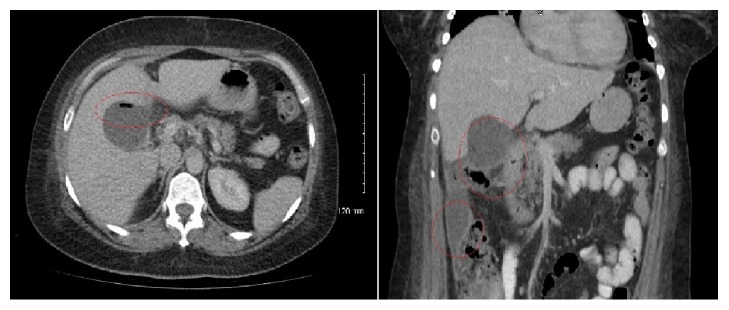
CT abdomen pelvis day 6: air within a distended gall bladder with persistent pericholecystic fat stranding, more marked than on prior imaging and extending to involve the hepatic flexure. New, peripherally enhancing fluid collection extending the inferior tip of the liver along the right paracolic gutter; appearances are suggestive of complicated cholecystitis with a gallbladder perforation.

She represented to the emergency department 48 hours after ERCP with severe right upper quadrant pain that radiated to the back. She was pyrexial and her white cell count was 20 × 10^9^/L and CRP was 83 mg/L. Liver function tests and urea and electrolytes were normal. A CT of the abdomen and pelvis showed periportal and pericholecystic fat stranding with hyperaemia of the extrahepatic bile duct with adjacent stranding (Figure 1). The findings were felt to be consistent with cholecystitis and ascending cholangitis and the patient was commenced on broad-spectrum antibiotics. She failed to settle with antibiotic therapy and the CRP rose to 330 mg/L and the white cell remained elevated at 19 × 10^9^/L. On day 6, a repeat CT abdomen demonstrated air within a distended gall bladder, with persistent pericholecystic fat stranding, extending to involve the hepatic flexure and a peripherally enhancing fluid collection in the right paracolic gutter (Figure 2). The findings were interpreted as complicated cholecystitis with a gallbladder perforation, possibly secondary to ERCP instrumentation. Emphysematous cholecystitis was not considered in the differential diagnosis at this point. An urgent surgical referral was sought and a radiological cholecystostomy tube was inserted at this point rather than emergent surgery. The biliary fluid was sent for culture and sensitivity. On day 7, the patient became increasingly short of breath and a chest X-ray showed a large right-sided pleural effusion and a smaller left-sided effusion. A pleural drain was inserted into the right side.

By day 10, there was limited clinical improvement and the patient was spiking intermittent temperatures. Repeat CT showed an interval reduction in the degree of pericholecystic fat stranding but persistent gallbladder distension and right paracolic collection. In addition, while there was an interval resolution of right pleural effusion, there was also a new interval development of right lower and middle lobe pneumonia. Bacterial culture and sensitivity results from the cholecystostomy biliary fluid were then returned urgently: this showed* Escherichia coli* and* Clostridium perfringens* bacteria, raising concern for a gangrenous gallbladder and emphysematous cholecystitis. Review of the CT scan from day 6 was made at this point and the findings were felt to be more consistent with emphysematous cholecystitis, rather than a gallbladder perforation as had been originally suggested. An emergent open cholecystectomy was performed and this confirmed the presence of a necrotic gallbladder with gas gangrene. The patient remained intermittently febrile for one week but this eventually settled with prolonged IV antibiotics. A repeat CT scan at day 10 after cholecystectomy showed almost complete interval resolution of the right paracolic gutter collection and the patient was discharged home on oral antibiotics on day 11 post-op. At 4 months after cholecystectomy the patient was doing well. Repeat CT of the abdomen and thorax showed resolution of the collections, a clear gallbladder bed, and some residual right basal atelectasis. Eight months after procedures, the abdominal pain has resolved.

## 3. Discussion

We present a case of acute emphysematous cholecystitis secondary to* Clostridium perfringens* infection following an ERCP in a young healthy woman. Acute emphysematous cholecystitis has been reported in association with a number of pathogens, including* Clostridium perfringens*,* E. coli,* and* B. fragilis*. Morbidity and mortality rates as high as 50% and 25%, respectively, have been described [1].* Clostridium perfringens* emphysematous cholecystitis is strongly associated with the presence of diabetes mellitus and older age and is more common in men [1–3].

Clostridial species constitute between 16 and 40% of total gut microbiota and are thought to play an important role in immune regulation within the gut [4].* Clostridium perfringens* is one of these commensals, although sepsis is rare in clinical practice. A relatively old study found* Clostridium perfringens* colonisation of the gallbladder in up to 19% of surgically removed gallbladders [5]. Whether this is reflective of rates of carriage in the normal population or whether* Clostridium perfringens* colonisation contributes to the development of gallbladder pathology necessitating surgery is not known.

Reports of* Clostridium perfringens* related emphysematous cholecystitis after ERCP are exceedingly rare, and to our knowledge there have only been 4 previously reported cases [6]. One of those four cases had similar features to the current case: a young female with no significant comorbidities who underwent an ERCP for treatment of possible Sphincter of Oddi Dysfunction which was complicated by* Clostridium perfringens* emphysematous cholecystitis requiring a cholecystectomy two days after ERCP [6]. The patient ultimately required a liver transplantation for secondary biliary cirrhosis. Given the rapid development of post-ERCP* Clostridium perfringens*-related emphysematous cholecystitis in these two patients with suspected FBSD, it is tempting to postulate that both were carriers of* Clostridium perfringens* within their gallbladders prior to the ERCP. Recently one case of post-ERCP gallbladder perforation was described. At the time of surgery several days later a gangrenous gallbladder was described but there was no information regarding the bacterial pathogens [7].

*Clostridium perfringens*-related emphysematous cholecystitis most commonly presents spontaneously as an acute cholecystitis type picture in elderly, acutely unwell patients, with RUQ pain, raised LFTs, and inflammatory markers [2, 8, 9]. Emphysematous cholecystitis is uncommon and can be difficult to distinguish clinically from typical acute cholecystitis, particularly early in the clinical course. Early CT imaging of the biliary tree is vital for early detection and diagnosis. Ultrasound is not a reliable modality to exclude emphysematous cholecystitis as evidenced by a report of* Clostridium perfringens* related emphysematous cholecystitis: ultrasound imaging of the gallbladder showed gallbladder wall thickening consistent with acute cholecystitis but CT imaging 2 hours later confirmed an emphysematous cholecystitis with perforation and pneumoperitoneum [2]. Plain X-ray imaging has detected radiological signs of EC [10, 11], but the sensitivity is very low.

Sepsis from* Clostridium perfringens* has been associated with other interventions of the biliary tree. One case report describes fulminant sepsis and death at 24 hours after liver biopsy secondary to* Clostridium perfringens* [12]. That patient was under investigation for recurrent cholangitis following previous cholecystectomy and had also undergone an ERCP prior to the liver biopsy.* Clostridium perfringens* sepsis was diagnosed post-mortem.

A high index of suspicion for* Clostridium perfringens* infection is required to ensure early detection and treatment of this potentially fatal infection. In this current case, there was a significant delay in the diagnosis of* Clostridium perfringens*-related emphysematous cholecystitis, which fortunately appears to have had no lasting sequelae for the patient. Air in the gallbladder was erroneously attributed to possible gallbladder perforation per-ERCP. The diagnosis of* Clostridium perfringens*-related cholecystitis was made 10 days after ERCP, whereas the imaging 6 days after the ERCP showed air in the gallbladder. Had there been a higher index of suspicion for emphysematous cholecystitis, despite the fact that the patient did not fit the “typical” demographic for* Clostridium perfringens*-related emphysematous cholecystitis (i.e., being older, being diabetic, and other comorbidities), the diagnosis could have been made earlier.

The diagnosis of FBSD can be difficult. The Rome IV consensus has recently reclassified the previous diagnoses of SOD types I, II, and III [13]. What was previously classified as SOD type I is now thought to represent papillary stenosis, a structural abnormality, and these patients should undergo biliary sphincterotomy. The EPISOD trial showed that patients with so-called Type III SOD do not respond to biliary sphincterotomy and ERCP should be avoided in these patients [14]. Those with previously designated SOD type II are now designated “suspected FBSD” and may benefit from biliary sphincterotomy. The diagnosis of FBSD is based on the presence of biliary type pain and either transient rises in liver enzymes or a dilated bile duct, but not both. ERCP is an invasive procedure and is associated with significant morbidity and mortality. The decision to proceed to ERCP in patients with suspected FBSD should not be taken lightly and the patient should have time to consider the possible complications [13]. The risks of ERCP must always be carefully weighed against potential benefits and noninvasive/medical treatments should be given an adequate trial before deciding to proceed to ERCP. In this case, the patient has been on a number of medications for over a year, without any benefit. A meta-analysis of post-ERCP complications showed a complication rate of 6.85% (95% CI 6.46–7.24%) with rates of 3.5%, 1.5%, 0.60%, and 0.33% respectively, for pancreatitis, infection, perforation, and death [15]. These rates of complication are even higher in the SOD subpopulation [13].

We herein describe the development of a serious, life-threatening* Clostridium perfringens*-related emphysematous, gangrenous cholecystitis in a previously healthy young woman, with suspected FBSD, who underwent a seemingly uneventful ERCP. This is the fifth such case to be described in the literature. One previously reported case also involved a young woman with possible SOD. It is interesting to postulate that functional biliary disorders such as FBSD and functional gallbladder disorder might be related to dysbiosis of the gallbladder/biliary tree.
